# HIV treatment and care services for adolescents: a situational analysis of 218 facilities in 23 sub‐Saharan African countries

**DOI:** 10.7448/IAS.20.4.21591

**Published:** 2017-05-16

**Authors:** Daniella Mark, Alice Armstrong, Catarina Andrade, Martina Penazzato, Luann Hatane, Lina Taing, Toby Runciman, Jane Ferguson

**Affiliations:** ^1^ Paediatric‐Adolescent Treatment Africa (PATA), Cape Town, South Africa; ^2^ Department of Psychology, University of Cape Town, Cape Town, South Africa; ^3^ Independent Consultant, London, United Kingdom; ^4^ HIV Department, World Health Organization, Geneva, Switzerland; ^5^ Health Adolescents & Young Adults Research Unit, Africa Health Research Institute, Mtubatuba, South Africa

**Keywords:** HIV, adolescent, treatment, care, service

## Abstract

**Introduction**: In 2013, an estimated 2.1 million adolescents (age 10–19 years) were living with HIV globally. The extent to which health facilities provide appropriate treatment and care was unknown. To support understanding of service availability in 2014, Paediatric‐Adolescent Treatment Africa (PATA), a non‐governmental organisation (NGO) supporting a network of health facilities across sub‐Saharan Africa, undertook a facility‐level situational analysis of adolescent HIV treatment and care services in 23 countries.

**Methods**: Two hundred and eighteen facilities, responsible for an estimated 80,072 HIV‐infected adolescents in care, were surveyed. Sixty per cent of the sample were from PATA's network, with the remaining gathered via local NGO partners and snowball sampling. Data were analysed using descriptive statistics and coding to describe central tendencies and identify themes.

**Results**: Respondents represented three subregions: West and Central Africa (*n* = 59; 27%), East Africa (*n* = 77, 35%) and southern Africa (*n* = 82, 38%). Half (50%) of the facilities were in urban areas, 17% peri‐urban and 33% rural settings. Insufficient data disaggregation and outcomes monitoring were critical issues. A quarter of facilities did not have a working definition of adolescence. Facilities reported non‐adherence as their key challenge in adolescent service provision, but had insufficient protocols for determining and managing poor adherence and loss to follow‐up. Adherence counselling focused on implications of non‐adherence rather than its drivers. Facilities recommended peer support as an effective adherence and retention intervention, yet not all offered these services. Almost two‐thirds reported attending to adolescents with adults and/or children, and half had no transitioning protocols. Of those with transitioning protocols, 21% moved pregnant adolescents into adult services earlier than their peers. There was limited sexual and reproductive health integration, with 63% of facilities offering these services within their HIV programmes and 46% catering to the special needs of HIV‐infected pregnant adolescents.

**Conclusions**: Results indicate that providers are challenged by adolescent adherence and reflect an insufficiently targeted approach for adolescents. Guidance on standard definitions for adherence, retention and counselling approaches is needed. Peer support may create an enabling environment and sensitize personnel. Service delivery gaps should be addressed, with standardized transition and quality counselling. Integrated, comprehensive sexual reproductive health services are needed, with support for pregnant adolescents.

## Introduction

Globally, an estimated 2.1 million adolescents (age 10–19 years) were living with HIV in 2013, of which 83% resided in sub‐Saharan Africa [1]. Due to the success of paediatric treatment and care, an increasing number of children living with HIV are surviving into adolescence. This success, however, brings novel challenges to health systems and HIV programmes required to respond to the unique needs of this changing cohort, with current HIV services generally geared towards adult or younger paediatric populations [2].

Adolescence is a period of transition marked by pubertal development, sexual identity formation and social and cognitive maturation. Negotiating these milestones can be both rewarding and challenging for all adolescents; for adolescents living with HIV (ALHIV), this transition is exacerbated by a chronic, stigmatized and sexually transmissible disease [3].

Age‐disaggregated programmatic data and evidence of effective service delivery interventions to support adolescents are lacking [4]. It is estimated that HIV‐related deaths among adolescents have tripled since 2000, making HIV the second leading cause of mortality in this age group worldwide [5]. Studies have indicated that adolescents have worse treatment outcomes [6], higher loss to follow‐up [7,8] (LTFU) and worse adherence [9,10] rates and higher risk of mental health disorders [11,12] than adults in various countries and contexts. Adolescents also present to antiretroviral (ART) services at later stages of disease progression and are at increased risk of death prior to starting ART [9].

The extent to which programmes are providing appropriate services to ALHIV is unknown. Recommendations are largely derived from privately funded, specialized treatment and care centres in urban settings [10], while in sub‐Saharan Africa, scale‐up of HIV services is largely through primary healthcare facilities. Improved understanding of decentralized ART services for adolescents is required to identify policy and programmatic gaps and requirements.

In September 2014, the World Health Organization (WHO) convened a consultation to review evidence on adolescent treatment and care. To support understanding of service availability for ALHIV, a South Africa‐based NGO undertook a facility‐level situational analysis of adolescent HIV treatment and care services in 23 sub‐Saharan African countries in July to August 2014. This manuscript describes the study's methodology and results, highlighting cross‐cutting service delivery barriers that constrain the treatment and care of ALHIV in sub‐Saharan Africa.

## Methods

This situational analysis utilized a cross‐sectional survey design with mixed methods (both quantitative and qualitative questions) developed through consultation with experts and stakeholders by Paediatric‐Adolescent Treatment Africa (PATA), formerly known as Paediatric AIDS Treatment for Africa. PATA's mission is to mobilize and strengthen a network of frontline health providers to improve paediatric and adolescent HIV treatment, care and support in sub‐Saharan Africa. The PATA network includes health providers at more than 300 associated health facilities that collectively care for over 200,000 children and adolescents on ART across 24 countries. The South Africa‐based non‐governmental organisation (NGO) provides technical capacity‐building opportunities to its network, as well as pilots and builds evidence around promising child‐ and adolescent‐friendly programming.

PATA developed two versions of its situational analysis survey: (1) a “high level” (HL) version with 31 items (Appendix 1) sent to facilities part of the PATA network and (2) a “deep dive” (DD) with 61 items (Appendix 2) sent to facilities with active PATA capacity‐building activities at the time. The surveys comprised yes/no, multiple‐choice (single and multiple answers) and open‐ended questions. All HL survey questions appeared in the DD version, with the latter having follow‐up questions to allow for more detailed responses, as well as additional questions on special issues such as adolescent transitioning support. Both surveys were administered in English, French and Portuguese and distributed to health facilities within the PATA network. Respondents at health facilities returned one questionnaire per facility to PATA as a Google Form or via fax or e‐mail in Microsoft Word from 15 July to 14 August 2014. Respondents comprised doctors (34%), nurses (21%), administrators (19%) and facility coordinators, counsellors, social workers or pharmacists (26%).

The HL was sent to 200 facilities and the DD to 58 facilities in PATA's network. Eight‐five facilities returned the former and 45 the latter. Snowball sampling [13,14] through PATA's network and local NGO partners (the Clinton Health Access Initiative, the South Saharan Social Development Organisation and the Elizabeth Glaser Pediatric AIDS Foundation (EGPAF)) expanded the study's reach, as respondents forwarded survey invitations to health providers working with ALHIV throughout sub‐Saharan Africa. This elicited a further 75 HL surveys. EGPAF additionally collected a further 13 DD surveys from facilities with active EGPAF programmes. Respondents primarily used Google Forms to return surveys, though some also returned completed questionnaires via e‐mail or fax.

Descriptive statistics were used to describe central tendencies. Following analysis and synthesis of survey data, prominent themes were identified and a coding structure established to systematically code and score open‐ended data according to a thematic analysis approach [15,16]. Three trained coders were subsequently provided illustrative examples and a scoring logic. Codes were summed and reported as percentages. Where ≥200 responses were available, results are displayed by region.

Ethics approval from an institutional review board (IRB​​​) was not obtained due to time constraints as the original purpose of this situational analysis was to inform the WHO Guidelines discussions at the adolescent treatment and care consultation held by the WHO in September 2014. Consent was obtained from respondents, who were informed that they could withdraw from the study at any time without penalty and that results would be presented as aggregated data without mention of specific facility names to preserve anonymity. Lastly, no patient‐linked data were collected to protect patient confidentiality.

## Results

**Figure 1:**
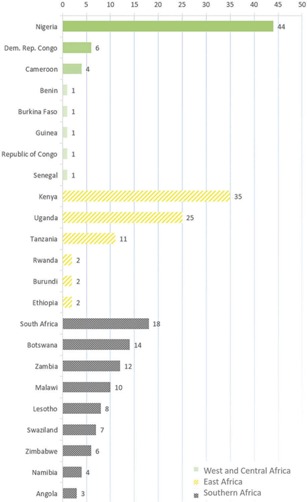
Locations of surveyed health facilities grouped according to three sub‐Saharan African subregions (*n* = 218).

Two hundred and eighteen health facilities from 23 countries returned surveys (Figure 1). Respondents represented three subregions: West and Central Africa (WCA) (*n* = 59; 27%), East Africa (*n* = 77, 35%) and southern Africa (*n* = 82, 38%). Half (50%) of the facilities were in urban areas, 17% peri‐urban and 33% rural. The estimated number of ALHIV in care at sampled facilities was 80,072.

The next sub‐sections detail data collected from the sample about the treatment and care of adolescents at their respective facilities. Results are organized according to the following themes: adolescent HIV treatment and care challenges; limited support of and data about adolescents as a patient population; treatment adherence; LTFU and retention in care; sexual and reproductive health (SRH) service integration; transition support; and early transition and special support for pregnant ALHIV. Unless otherwise stated, the number of respondents for the HL or DD survey is noted in the heading of each sub‐section. Questions asked in both the HL and the DD surveys are labelled only under the former.

### Adolescent HIV treatment and care challenges (HL, *n* = 208)

​​​Health providers most commonly (40%) cited non‐adherence and treatment failure as their most prominent challenge in initiating treatment, caring for and retaining adolescents (Table 1). The second was non‐disclosure of HIV status (30%), including non‐disclosure to the adolescent and onward non‐disclosure. Socioeconomic barriers ‐ such as poverty, transportation costs and food insecurity ‐ were the third most commonly cited challenge (25%) (Box 1).

**Table:** Box 1. Health provider quotations that are representative of the most prominent challenges in providing treatment and care for adolescents

*“Most of the adolescents do not comply with treatment because they are in their self‐discovery stage where they start questioning.”* Nurse Swaziland
*“Many guardians try as much as possible to delay disclosure because they do not want to hurt/offend the child.”* Doctor Malawi
*“Over 50% of our adolescents are orphans lacking in all basic needs.”* Doctor Malawi

**Table 1:** Treatment and care challenges at 208 health facilities in sub‐Saharan Africa​​​

	Non‐adherence/treatment failure (%)	Non‐disclosure (%)	Socioeconomic barriers (%)	Stigma, myths and traditional medicine (%)	Poor caregiver support and orphanhood (%)	Loss to follow‐up (%)	Sexual and reproductive health (%)
At treatment initiation	34	37	18	20	27	13	2
During long‐term care	48	38	28	30	17	22	16
Ensuring retention in care	38	14	30	17	16	23	11
Summary percentage across all areas	**40**	**30**	**25**	**22**	**20**	**19**	**10**

### Limited support of and data around adolescents as a patient population

#### Definition of adolescence (HL, n = 215)

Approximately one‐third of facilities (35%) reported attending to adolescent patients separately from adult and/or paediatric patients. In this case, most (88%) had a day or time to attend exclusively to adolescents, while some offered adolescents dedicated staff (10%) or spaces (8%).

**Figure 2:**
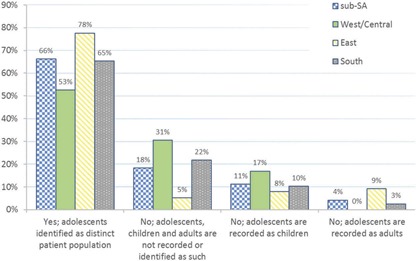
Regional breakdown of adolescents identified as a distinct patient population in or from sub‐Saharan African health facility records (*n* = 215).

Interestingly, 26% of facilities did not have an official working definition of adolescence. Their definitions also varied greatly, ranging from 8 to 21 years. Figure 2 shows a regional breakdown of age disaggregation of facility records, with WCA countries reporting the lowest levels of identifying adolescents as a distinct patient population. Two‐thirds of the sample (66%) reported maintaining either up‐to‐date paper or electronic records in which adolescents could easily be distinguished from children (1–9 years) and adults (<18 years).

#### Descriptors in records (HL, n = 214)

**Figure 3:**
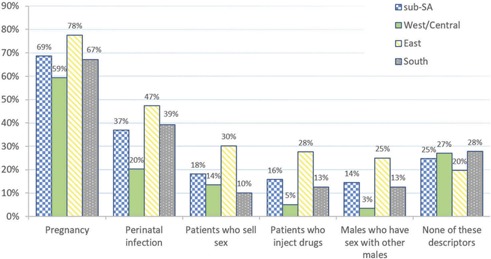
Regional breakdown of descriptors recorded about adolescent patients at sub‐Saharan African health facilities (*n* = 214).

Figure 3 presents descriptors related to clinical issues or key population status that health providers used in ALHIV patient records. Twenty‐five per cent of facilities across all regions did not record any descriptors, with those in WCA least likely to capture this information. Young key population status (such as young people who sell sex, young people who inject drugs, young men who have sex with men and young transgender people) was recorded in adolescent records at 14–18% of facilities, depending on the specific status.

#### Treatment outcomes monitoring (HL, *n* = 214)

**Figure 4:**
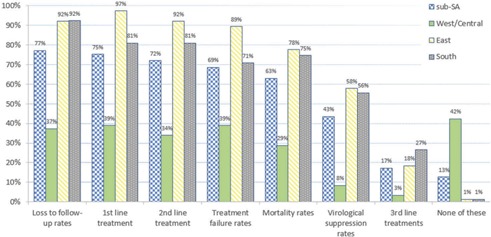
Regional breakdown of treatment outcomes monitored by sub‐Saharan African health facilities (*n* = 214).

Virological suppression was monitored in less than half (43%) of facilities (Figure 4). Eighty per cent of facilities that recorded treatment outcomes did not disaggregate by age. East African facilities reported better monitoring of treatment outcomes. Nearly half (42%) of the respondents in WCA reported that none of these treatment outcomes were being monitored. Fifty‐six DD respondents (97%) cited ongoing lack of adherence to prescribed treatment regimens as the primary reason for needing to transition adolescents from first‐ to second‐line treatment.

### Treatment adherence (HL, n = 216)

Adherence counselling was reportedly offered at 87% of facilities surveyed. Health providers said that they predominantly gave general explanations for needing to adhere to ART regimens during counselling sessions with adolescents. They also discussed the negative implications of non‐adherence, such as the development of drug resistance. Discussions about repercussions, however, sometimes took the form of scaremongering, as demonstrated by a nurse in Namibia who noted that health providers *“counsel on the danger of resistance and the scare of opportunistic infections as well as death.”*

Two‐thirds (67%) of facilities reported offering other services to improve treatment adherence in adolescents. The most common, provided by 49% (*n* = 145) of facilities, were peer support, activities and clubs. Based on additional questions that explored perceived effectiveness of intervention, most facilities identified peer services as the most effective adherence intervention (DD, *n* = 43), but no facility was able to provide supporting evidence. One doctor in Ethiopia, however, did note, *“peer to peer youth group[s] are highly effective since a common bond forms and adolescents share their own personal issues and find solutions for common problems through open discussion without fear of discrimination.”*

More than a third (39%) of respondents (DD, *n* = 56) reported having no guidelines or protocols for managing adolescents with adherence challenges. Forty‐five per cent (DD, *n* = 55) reported no mechanism for assessing adherence, and no cut‐off for determining non‐adherence.

### LTFU and retention in care (*n* = 216)

Most facilities define LTFU as being three or more months since an adolescent's last facility visit (66%). When asked if they provide support services to ensure adolescents are retained in care, less than two‐thirds (61%) answered positively to offering such services. Forty‐one per cent reported having no guidelines or protocols for managing adolescents with retention challenges.

Where retention support was offered to adolescents, the two most common approaches were peer support, activities and clubs (34%, *n* = 132) and home visits (31%). In response to an open‐ended question, most facilities (DD, *n* = 37) reported anecdotally that these two approaches are the most effective retention support activities.

### SRH service integration (HL, *n* = 217)

Sixty‐three per cent reported providing SRH services for adolescents. Where offered, SRH services most frequently included family planning and contraceptive distribution (72%). Less than half providing these services offered general counselling (40%), sexually transmitted infection screening and treatment (31%), cervical cancer screening (14%), prevention of mother‐to‐child transmission (PMTCT) or antenatal care (ANC) (10%), or voluntary medical male circumcision (10%).

### Transition support for adolescents (DD, *n* = 54)

Sixty‐three per cent provided counselling to support adolescent transition from paediatric to adult services, and just over half (51%) reported having guidelines or protocols for this process. The age at which transition occurs varies greatly, with the most common being transition from paediatric services at 18 years (23%, *n* = 43), followed by 10 (14%), 12 (12%) and 15 (12%) years.

Nearly a third (38%, *n* = 34) of respondents said they offer transition counselling routinely or for more than five sessions (Table 2). Those who offered routine counselling described the frequency and duration of routine counselling as being based upon an individual's needs and conducted either on a one‐to‐one basis and/or in a group setting. Seventeen facilities said the duration of these counselling sessions was either ≤15 min (29%, *n* = 17) or more likely ≥15 min (71%).

**Table 2:** Provision of counselling to support adolescent transition from paediatric to adult services – frequency, personnel involvement and counselling content

Frequency of counselling before adolescent transition from paediatric to adult services		
Frequency/number of sessions	Number of facilities	Percentage of facilities
Routine or >5	13	38
1	2	6
2	3	9
3	8	24
4	4	12
Unspecified	4	12
*Total number of responses (n)*	*34*	‐
**Involvement of facility personnel in adolescent transitioning process**		
**Facility personnel or persons**	**Number of facilities**	**Percentage of facilities**
Counsellor	19	59
Doctor/clinician	14	44
Nurse	12	38
Caregiver	10	31
Social worker	8	25
Community health worker	4	13
Peer supporter	2	6
Pharmacist or pharmacy technician	1	3
Psychologist	1	3
Other staff	4	13
*Total number of responses (n)*	*32*	‐
**Transition counselling content**		
**Counselling content**	**Number of facilities**	**Percentage of facilities**
Transition process	14	44
Sexual and reproductive health	9	28
Adherence	6	19
Benefits of health habits/lifestyle	6	19
Emotional well‐being and resilience	5	16
Disclosure support	5	16
Socioeconomic and livelihood guidance	2	6
Life and psychosocial skill guidance	2	6
Social support access	1	3
Routine counselling benefits	5	16
*Total number of responses (n)*	*32*	‐

Table 2 also outlines the various persons involved in adolescent transitioning by facility.

Eight facilities further elaborated when they generally commence the transitioning process. Most (38%, *n* = 8) started approximately six months prior to adolescents being moved to adult services. The time range for transitioning commencement ranged from three months to three years prior.

In terms of facility personnel, counsellors tended to be most involved (59%, *n* = 19) in adolescent transitioning processes, followed by doctors/clinicians (44%), nurses (38%) and social workers (25%). Caregivers are involved at 31% of facilities.

Additionally, Table 2 presents cumulative totals of content health providers discussed during ALHIV transition counselling. Thirty‐two respondents described the content discussed during transitioning counselling. Forty‐four per cent (*n* = 32) reported that counselling during the transition period tends to focus on the process of moving adolescent patients’ treatment and care from a paediatric to adult facility.

### Early transition and special support for pregnant ALHIV (DD, *n* = 57)

Transition for pregnant adolescents varied amongst facilities, with 12 (21%) reporting that pregnancy led to an adolescent's early transition to adult PMTCT/ANC and ART clinics. A nurse in Namibia explained, “*The reason why we move them is we try to discourage unwanted pregnancies. We counsel them before they are pregnant that there are two ways to graduate to adult group, by age and falling pregnan[t]…”* Only two facilities said they transitioned young mothers back to adolescent clinics post‐pregnancy. A doctor from one of these facilities in Uganda said these young mothers are cautioned *“not to mess up again but concentrate on studies.”*

Support to HIV‐infected pregnant adolescents also appears to be limited: less than half (46%) reported catering to their special needs, such as offering PMTCT, ANC, special case management, support groups for pregnant ALHIV, general counselling, social services and/or grant referrals.

## Discussion

The results of this situational analysis point to an insufficiently targeted approach for adolescents and highlight five key challenges that must be addressed.

### Non‐adherence, LTFU and insufficient support mechanisms

Facilities highlighted poor adherence as their key challenge. This is consistent with literature [17], including a recent global consultation of young people living with HIV [18] that reported significant adherence barriers adolescents face on treatment. Moreover, facilities reported insufficient protocols for determining and managing both non‐adherence and LTFU, as well as limited use of counselling tools to inform their interventions. This is problematic as research indicates that adolescents and young people are at high risk of LTFU, especially those aged 15–19 years [7,8,19,20].

Some facilities indicated peer support as the most effective intervention to address adherence and retention issues, but were unable to provide supporting evidence. Recent data from Zimbabwe indicate that adolescent‐led community‐based peer interventions improve linkage to care, adherence, retention and psychosocial well‐being in ALHIV [21]. This thus adds to a small body of evidence [22,23] that peer interventions create opportunities for shared experiences and provide a source of support [18] for ALHIV.

### Service delivery gaps: optimizing adolescent care and transition

While most facilities reported attending to adolescents with adults and/or children, some did allocate a specific time to focus on adolescents. Experience suggests that in many settings, especially primary health care settings, separate services for adolescents may not be feasible.

Transition was process‐oriented, with counselling provided only by some facilities. Implementing a standardized transition approach with high‐quality, developmentally appropriate counselling is important, with special attention to address the needs of pregnant adolescents. Our assessment indicates that pregnant adolescents may be forced to transition abruptly out of adolescent or paediatric services without adequate counselling or support and may face stigma from health providers and peers. This is an emerging issue, with studies showing higher risk of MTCT and greater likelihood of LTFU among HIV‐infected adolescent mothers compared to adult mothers [24–26]. Pregnant adolescents should continue to receive targeted support and adolescent‐friendly health services [27] in a non‐stigmatizing environment.

### Poor integration of SRH services

In addition to early transitioning of pregnant adolescents, SRH services are not being offered comprehensively and are often limited to family planning and contraceptives. These findings reinforce reports from adolescent consultations and qualitative studies of there being a disparity between adolescent needs and the limited information and services they receive [18,28–30]. This requires urgent attention, given the importance of these services in preventing new infections and unwanted pregnancies. Integrated, comprehensive SRH services are critical and should be provided in an enabling and supportive setting. An appropriate package of SRH services that meets the needs of ALHIV should be offered within facilities and through referrals.

### Insufficient data disaggregation and outcomes monitoring

As UNICEF recently found [31], limited ability to identify adolescents as a sub‐population at facility level remains a significant challenge. Facilities are not capturing key descriptors required to address the special needs of adolescent sub‐groups, such as mode of infection and key population information. Finally, outcomes data are not being well recorded for adolescents. Inadequate adoption of standardized age definitions, data disaggregation and outcomes monitoring in cohort and surveillance data limit our understanding of adolescent service gaps, as well as the degree to which guidelines and protocols can be specified, implemented and monitored [32]. Until this is addressed, it will be difficult to conduct, plan and monitor adolescent treatment and care and undertake operational research required to improve service quality for ALHIV.

### Limitations

There are several limitations to our analysis. First, the results of this situational analysis may not be sufficiently representative. A third (33%) of the sample was from rural areas, and we did not undertake controlled random sampling. Second, the PATA network, itself a capacity‐building platform, may have led to a positive bias in that PATA network facilities might provide better services for adolescent patients. Third, all data are based on reports from healthcare staff. Further on‐site validation or with other stakeholders (including adolescent patients themselves) was not possible, given the scope of the study, as well as time and resource limitations.

## Conclusions

This multi‐country situational analysis provides key insights into the status of HIV treatment and care services for adolescents in sub‐Saharan Africa. Overall, the analysis highlighted a wide variety of approaches in the region. Additionally, it flags critical areas for research and intervention in adolescent adherence to ART and engagement in care from the perspectives of frontline health providers from 23 sub‐Saharan countries.

Further implementation research is needed to identify which service delivery models are most effective in different contexts to improve ALHIV adherence, retention and treatment outcomes. Moreover, data gathering, analysis and reporting systems will need to be strengthened across sub‐Saharan Africa in order to better monitor HIV‐infected adolescents and the effectiveness of these approaches to improve quality, uptake and impact of adolescent HIV services. New initiatives to address the urgent needs of the growing adolescent population must be put in place to reach global treatment targets.

## Competing interests

The authors have no competing interests to declare.

## Acknowledgements

The authors thank the following individuals for their support and feedback: Ronnie Lovich (EDC); Shaffiq Essajee (WHO); Nandita Sugandhi (CHAI); Anouk Amzel (USAID); Eric Dziuban (CDC); Carrie Foti (EDC); Kate Iorpenda (International HIV/AIDS Alliance); Ed Ngoksin (GNP+/Y+); Neil Gupta (PIH); Lucie Cluver, Elona Toska & Beth Vale (University of Oxford); Elizabeth Obimbo (University of Kenya); and Sabrina Kitaka (Makerere University). Lastly, we acknowledge health facility staff who contributed their time and insights to this situational analysis.

To access the supplementary material to this article please see http://doi.org/10.7448/IAS.20.4.21591 under Article Tools online.

## Disclaimer

This manuscript represents the views of the authors, and the findings and conclusions included here do not necessarily represent the views of the World Health Organization.

## Abbreviations

ALHIV, adolescents living with HIV; ANC, antenatal care; ART, antiretroviral treatment; CHAI, Clinton Health Access Initiative; DD, deep‐dive; EGPAF, Elizabeth Glaser Paediatric AIDS Foundation; HL, high level; LTFU, lost to follow‐up; NGO, non‐governmental organisation; PMTCT, prevention of mother‐to‐child transmission; SRH, sexual and reproductive health; SSDO, South Saharan Social Development Organization; WCA, West and Central Africa; WHO, World Health Organization.

## Supporting information

Appendix B: Deep‐dive survey

Appendix A: High‐level survey
