# Supplementary material for: HIV treatment and care services for adolescents: a situational analysis of 218 facilities in 23 sub‐Saharan African countries
Source: J Int AIDS Soc. 2017 May 16;20(Suppl 3):21591. doi: 10.7448/IAS.20.4.21591 (PMC5719719; doi:10.7448/IAS.20.4.21591)
Supplement: Supplementary file 1 — Appendix B: Deep‐dive survey [file JIA2-20-21591-s001.docx]

**Appendix A: High-level survey**


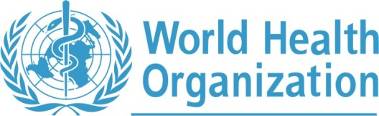

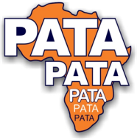


**Clinic information:**

| Country: |  |
| --- | --- |
| Clinic name: |  |
| Respondent name & surname: |  |
| Respondent email: |  |
| Position in the clinic: |  |
| Rural/peri-urban/urban |  |

How does **your clinic** define the age ranges below?

|  | From: | To: |
| --- | --- | --- |
| Childhood (Incl. adolescence) | 0 |  |
| Adulthood |  | 100 |

Does **your clinic** have a **working definition of adolescence**?

If yes, please describe the age range:

| From: | To: |
| --- | --- |
|  |  |

**If No**, please answer the rest of the survey using the **WHO definition of 10–19 years old** when responding.

**Adolescent HIV treatment and care information:**

1. What are the **three biggest challenges** your clinic faces in **initiating** HIV treatment and care to adolescents?

|  |
| --- |

1. What are the **three biggest challenges** your clinic faces when **dealing with** HIV+ adolescents **in treatment**?

|  |
| --- |

1. What are the **three biggest challenges** your clinic faces in **sustaining** HIV treatment and care to adolescents?

|  |
| --- |

1. Does your clinic offer **separate HIV treatment and care services for adolescents** or are they combined with children or adults? For example: Special clinic days, times or venues

If yes, please describe:

|  |
| --- |

If other, please describe:

|  |
| --- |

1. Does your clinic **register** have a way **to identify or record** HIV+ adolescent patients?

1. Does your clinic **record any of the following information** about HIV+ adolescents? Please select all of those that apply.

1. Please select the **HIV treatment outcomes data** that your clinic **currently captures**. Please select all of those that apply.

1. Of the **above treatment outcomes data** that your clinic does capture, **is this done specifically for adolescents**?

1. What does your **clinic do with the treatment outcomes data** that you capture? Please select all of those that apply.

If other, please describe:

|  |
| --- |

1. Does your clinic offer **adherence counselling** to HIV+ adolescents?

If yes to any of these, please describe the content of counselling:

|  |
| --- |

1. Does your clinic offer **any other support or services** to ensure **treatment adherence** for HIV+ adolescents?

If yes, please describe:

|  |
| --- |

1. Does your clinic offer any **support or services** to ensure **retention in care** for HIV+ adolescents?

If yes, please describe:

|  |
| --- |

1. Does your clinic offer **sexual and reproductive health services** to HIV+ adolescents? If yes, please describe which services are provided and where they are offered.

If yes, please describe:

|  |
| --- |

Thank you for your time!
