# Supplementary material for: HIV treatment and care services for adolescents: a situational analysis of 218 facilities in 23 sub‐Saharan African countries
Source: J Int AIDS Soc. 2017 May 16;20(Suppl 3):21591. doi: 10.7448/IAS.20.4.21591 (PMC5719719; doi:10.7448/IAS.20.4.21591)
Supplement: Supplementary file 2 — Appendix A: High‐level survey [file JIA2-20-21591-s002.docx]

**Appendix B: Deep-dive survey**


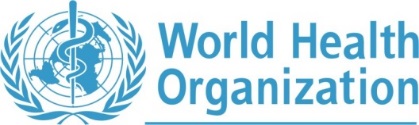

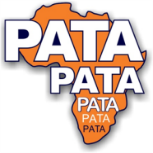


**Clinic information:**

| Country: |  |
| --- | --- |
| Name of clinic: |  |
| Respondent name & surname: |  |
| Respondent email: |  |
| Position in the clinic: |  |
| Rural/peri-urban/urban |  |

How does **your clinic** define the age ranges below?

|  | From: | To: |
| --- | --- | --- |
| Childhood | 0 |  |
| Adulthood |  | 100 |

Does **your clinic** have a **working definition of adolescence**?

If yes, please describe the age range:

| From: | To: |
| --- | --- |
|  |  |

**If No**, please answer the rest of the survey using the **WHO definition of 10–19 years old** when responding.

**Adolescent HIV treatment and care information:**

1. What are the **three biggest challenges** your clinic faces when **caring for** HIV+ adolescents in treatment?

|  |
| --- |

1. What are the **three biggest challenges** your clinic faces in **initiating** HIV treatment and care to adolescents?

|  |
| --- |

1. What are the **three biggest challenges** your clinic faces in **sustaining** HIV treatment and care to adolescents?

|  |
| --- |

1. Does your clinic offer **separate HIV treatment and care services for adolescents** or are they combined with children or adults? For example: Special clinic days, times or venues

If yes, please describe:

|  |
| --- |

If other, please describe:

|  |
| --- |

1. Does your clinic **register** have a way **to identify or record** HIV+ adolescent patients?

1. Does your clinic **record any of the following information** about HIV+ adolescents? Please select all of those that apply.

1. Please select the **HIV treatment outcomes data** that your clinic **currently captures**. Please select all of those that apply.

1. Of the **above treatment outcomes data** that your clinic does capture, **is this done specifically for adolescents**?

1. What does your **clinic do with the treatment outcomes data** that you capture? Please select all of those that apply.

If other, please describe:

|  |
| --- |

1. Does your clinic provide **viral load monitoring** of HIV+ **adolescents**?

If yes, please describe how often:

|  |
| --- |

1. Does your clinic **define viral load failure**?

If yes, please describe:

|  |
| --- |

If yes, please describe what **procedures you follow once failure has been confirmed**:

|  |
| --- |

1. What are the **key challenges** that you face when switching an adolescent HIV+ patient **to 2^nd^ line treatment**? Please select all that apply.

If other, please describe:

|  |
| --- |

1. What are the most **common or frequent clinical presentations** seen amongst HIV+ adolescents? Please select all of those that apply.

If other, please describe:

|  |
| --- |

1. Please could you fill in the **number of adolescent patients** that your clinic treats for each outcome below. If not applicable, please write **‘not applicable’** in the box provided.

| Number of adolescent patients? | Number of adolescent patients on antiretroviral treatment? | Rate of loss to follow up in adolescent patients? |
| --- | --- | --- |
|  |  |  |
| Number of adolescent patients on 1st line treatment? | Number of adolescent patients on 2nd line treatment? | Number of adolescent patients on 3rd line treatment? |
|  |  |  |

1. How does your clinic **define treatment adherence**? Please describe.

|  |
| --- |

1. Does your clinic have **guidelines or protocols** in place for managing adolescents who are facing **adherence** **challenges**?

If yes, please describe:

|  |
| --- |

1. Does your clinic offer **adherence counselling** to HIV+ adolescents?

If yes, please describe **the content of counselling**:

|  |
| --- |

If yes, please describe **who is involved** in these **adherence counselling** sessions:

|  |
| --- |

1. Does your clinic offer **any other support or services** to ensure **treatment adherence** for HIV+ adolescents?

If yes, please describe:

|  |
| --- |

Which of the **above approaches is most effective** and **why**?

|  |
| --- |

Have you ever **measured** the **effectiveness** of the above? And if so, **how**?

|  |
| --- |

1. How does your clinic **define loss to follow-up**? Please describe.

|  |
| --- |

1. Does your clinic have **guidelines or protocols** in place for managing adolescents who are facing **retention** **challenges**?

If yes, please describe:

|  |
| --- |

1. Does your clinic offer any **support or services** to ensure **retention in care** for HIV+ adolescents?

If yes, please describe:

|  |
| --- |

Which of the **above services are most effective** and **why**?

|  |
| --- |

Have you ever **measured** the **effectiveness** of the above? And if so, **how**?

|  |
| --- |

1. What does your clinic do to **track** HIV+ adolescents who have been **lost to follow-up** and **bring them back** **into** **care**? Please describe.

|  |
| --- |

1. At what age are patients moved out of paediatric services (if applicable)?

|  |
| --- |

1. When a **child leaves paediatric HIV services** (if applicable), are they offered any **counselling** about this move to **new services**? If yes, please state the number of sessions offered and how long they last. For example, 3 sessions of 15 minutes.

If yes, please describe:

Frequency Duration

|  |  |
| --- | --- |

1. What is the **content of counselling given during transition** (if applicable), and how does this differ from the content of counselling given during non-transition times?

|  |
| --- |

If yes, please describe **who is involved** in these **transitioning counselling** sessions:

|  |
| --- |

1. Does your clinic have **guidelines** in place that outline the **process for transition**?

1. If an **adolescent becomes pregnant**, is she moved into adult services **sooner**? How is this done?

If yes, please describe the process:

|  |
| --- |

1. Does your clinic manage the **special needs of pregnant adolescents**, and if so **how**?

If yes, please describe:

|  |
| --- |

1. Does your clinic offer **sexual and reproductive health services** to HIV+ adolescents? If yes, please describe which services are provided and where they are offered.

If yes, please describe:

|  |
| --- |

1. To **what extent are other health-related services integrated** into HIV treatment and care services? For example these could include nutrition, counselling, skills training.

1. Does your clinic have **relationships or referral systems** with any of the below **community structures** that provide **additional support or complementary services** for HIV+ youth? Please select all that apply.

If CBO, please describe the nature and/or the structure of this relationship:

|  |
| --- |

If FBO, please describe the nature and/or the structure of this relationship:

|  |
| --- |

If NGO, please describe the nature and/or the structure of this relationship:

|  |
| --- |

If Other, please describe the nature and/or the structure of this relationship:

|  |
| --- |

Thank you for your time!
